# Covalent NEDD8 Conjugation Increases RCAN1 Protein Stability and Potentiates Its Inhibitory Action on Calcineurin

**DOI:** 10.1371/journal.pone.0048315

**Published:** 2012-10-31

**Authors:** Eun Hye Noh, Hee Sook Hwang, Hee Sun Hwang, Boram Min, Eunju Im, Kwang Chul Chung

**Affiliations:** Department of Systems Biology, College of Life Science and Biotechnology, Yonsei University, Seoul, Korea; German Cancer Research Center, Germany

## Abstract

Similar to ubiquitin, regulatory roles for NEDD8 (neural precursor cell-expressed developmentally down-regulated 8) are being clarified during cell growth, signal transduction, immune response, and development. However, NEDD8 targets and their functional alterations are not well known. *Regulator of calcineurin 1* (*RCAN1/DSCR1P1*) is located near the Down syndrome critical region on the distal part of chromosome 21, and its gene product is an endogenous inhibitor of calcineurin signaling. RCAN1 is modified by ubiquitin and consequently undergoes proteasomal degradation. Here we report that NEDD8 is conjugated to RCAN1 (RCAN1-1S) via three lysine residues, K96, K104, and K107. Neddylation enhances RCAN1 protein stability without affecting its cellular location. In addition, we found that neddylation significantly inhibits proteasomal degradation of RCAN1, which may underlie the ability of NEDD8 to enhance RCAN1 stability. Furthermore, neddylation increases RCAN1 binding to calcineurin, which potentiates its inhibitory activity toward downstream NFAT signaling. The present study provides a new regulatory mechanism of RCAN1 function and highlights an important role for diverse RCAN1-involved cellular physiology.

## Introduction

The gene *Regulator of calcineurin 1* (*RCAN1;* also called as *DSCR1, Adapt78, MCIP1* or *calcipressin 1*) is located on chromosome 21, near the Down syndrome critical region [1]. It is highly expressed in the brain, heart, and skeletal muscles of Down syndrome patients [2], and interacts physically and functionally with the Ca^2+^/calmodulin-dependent protein phosphatase, calcineurin [3]. Based on two controversial findings, RCAN1 has been proposed to be a feedback inhibitor of calcineurin. First, although RCAN1 overexpression suppresses calcineurin signaling [3], [4], calcineurin activity is greatly diminished in the hearts of RCAN1-knock-out mice [5]. The nuclear factors of activated T cells (NFATs) are a family of transcription factors that transduce calcium signals in the immune, cardiac, muscular, and nervous systems [6]. Calcineurin dephosphorylates multiple serine residues in the N-terminal of various NFAT proteins. This dephosphorylation causes translocation from the cytoplasm to the nucleus, where they engage a variety of transcription factors and activate calcineurin-responsive genes [7].

The RCAN1 gene consists of seven exons plus an alternative first one (exon 1 through 4) [2]. There are four possible transcripts but the major transcriptional products are isoforms that include exon 1 (RCAN1-1) or 4 (RCAN1-4). RCAN1-1 encodes a protein of 197 amino acids and is abundant primarily in fetal and adult brains [2]. A recent study revealed an additional start site upstream of exon 1, which produces RCAN1-1 with 252 amino acids [8]. In order to avoid confusion between these two products, the former is referred as RCAN1-1S (short form) and the latter as RCAN1-1L (long form).

NEDD8 (neural precursor cell-expressed developmentally down-regulated gene 8) is a small ubiquitin-like protein that shares 60% sequence identity and 80% homology with ubiquitin [9]. NEDD8 is conjugated to substrate proteins in a process known as neddylation. The carboxy-terminal glycine 76 residue of NEDD8 forms a thioester bond with APPBP1-Uba3, the E1 enzyme. Activated NEDD8 then transfers to Ubc12, the E2 enzyme [10], [11]. Ultimately, E2 loaded with NEDD8 binds a substrate protein lysine residue and forms an isopeptide bond by E3 ligase. RING box protein-1 (RBX1) is a RING component of the SCF ubiquitin ligase complex composed of Skp-1, Cullin and F-box proteins. RBX1 is a NEDD8 E3 ligase for cullins [12]. Neddylation has been reported to play important roles in cell growth, embryogenesis, and development. Currently, there are less than ten reported NEDD8 targets, including the cullin family, the Von-Hippel Lindau tumor suppressor (pVHL), p53, and Mdm2 [11], [13]. Although the effect of neddylation is largely dependent upon the individual target, it can be classified into three general and direct effects [11], [13]. First, neddylation induces conformational changes of the targets. Second, it can prohibit certain interactions due to the conformational changes. Finally, neddylation provides a binding surface and stimulates recruitment of other NEDD8-interacting partner.

We previously reported that the RCAN1 (RCAN1-1S) protein is covalently modified by ubiquitin and subsequently processed by the ubiquitin-proteasome system (UPS) [14]. Based on this finding, we were interested whether RCAN1 is modified by interaction with other small ubiquitin-like modifiers and how it might affect RCAN1 biochemical and functional activity. In this study, we focus on NEDD8 modification of RCAN1. Our results reveal that covalent NEDD8 attachment regulates RCAN1 biochemical properties and subsequently affects calcineurin activity and NFAT signaling.

## Results

### RCAN1 is a Target of Covalent NEDD8-conjugation

**Figure 1:**
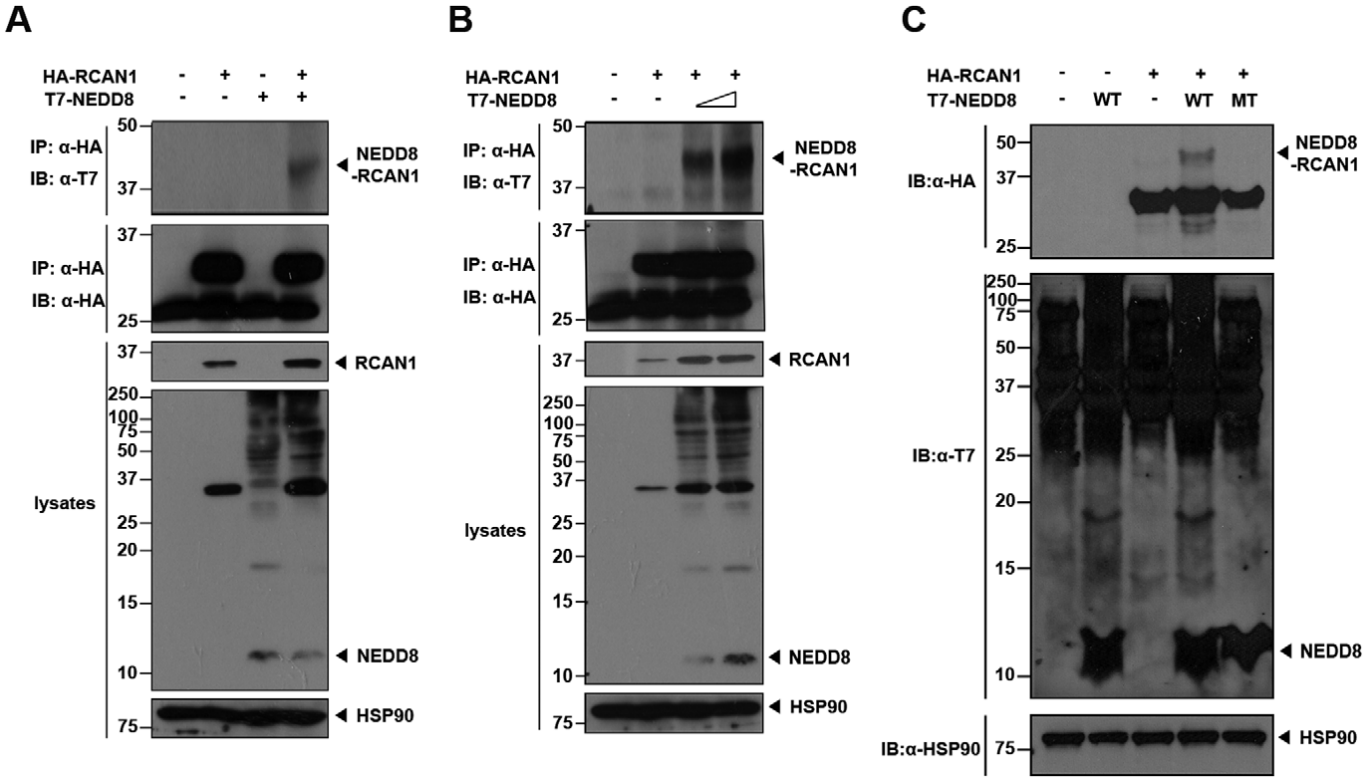
RCAN1 is neddylated in HEK293 cells. (**A**) HEK293 cells were transfected for 24 h with HA-tagged RCAN1 and/or T7-tagged NEDD8 plasmids. Immunoprecipitation (IP) was performed with HA antibodies, and the immunocomplexes were analyzed by western blotting with T7 antibodies. The HSP90 antibody is used as a loading control. (**B**) After transfection for 24 h with HA-RCAN1 alone or together with increasing doses of T7-NEDD8, immunoprecipitation was performed with HA antibodies followed by western blotting with T7 antibodies. (**C**) HEK293 cells were transfected for 24 h alone or in combination with HA-RCAN1, T7-tagged wild-type NEDD8, or its conjugation-defective mutant (MT). Cells were lysed with lysis buffer containing 8 M urea, and lysates were analyzed by immunoblot with HA antibodies.

To investigate whether RCAN1 can be covalently modified with NEDD8 in mammalian cells, HEK293 cells were transfected with plasmids encoding HA-tagged RCAN1 alone or together with either T7-tagged NEDD8 or its conjugation-defective mutant (NEDD8-ΔGG). This mutant lacks the essential C-terminal glycine-glycine residues. After cells were lysed, NEDD8-conjugation to RCAN1 was evaluated by immunoprecipitating ell extracts with HA antibodies, followed by immunoblotting with the T7 antibody. As shown in Figure 1A, an upper-shifted form of RCAN1 was observed in cells co-transfected with HA-RCAN1 and wild-type T7-NEDD8. The size of this band (∼41 kDa) corresponds to mono-neddylated RCAN1 protein and was not detected in cells transfected with NEDD8 or HA-RCAN1 alone, or cells co-transfected with both HA-RCAN1 and NEDD8-ΔGG (Fig. 1A). In addition, when cells were co-transfected with HA-tagged RCAN1 and increasing amounts of T7-NEDD8, the amount of NEDD8-conjugated RCAN1 protein increased in a dose-dependent manner (Fig. 1B). Next, we treated cells with lysis buffer containing 8 M urea and evaluated whether the covalently NEDD8-modified RCAN1 band is still detected. This eliminates the possibility that RCAN1 indirectly binds to other intracellular neddylated proteins. Western blot analyses with HA antibodies detects the same mono-neddylated RCAN1 band, as observed in the co-immunoprecipitation assays, as well as the unmodified RCAN1 protein (Fig. 1C). These results suggest that RCAN1 may be a target of neddylation in mammalian cells.

### Mapping of NEDD8-targeting Site(s) within the RCAN1 Protein

**Figure 2:**
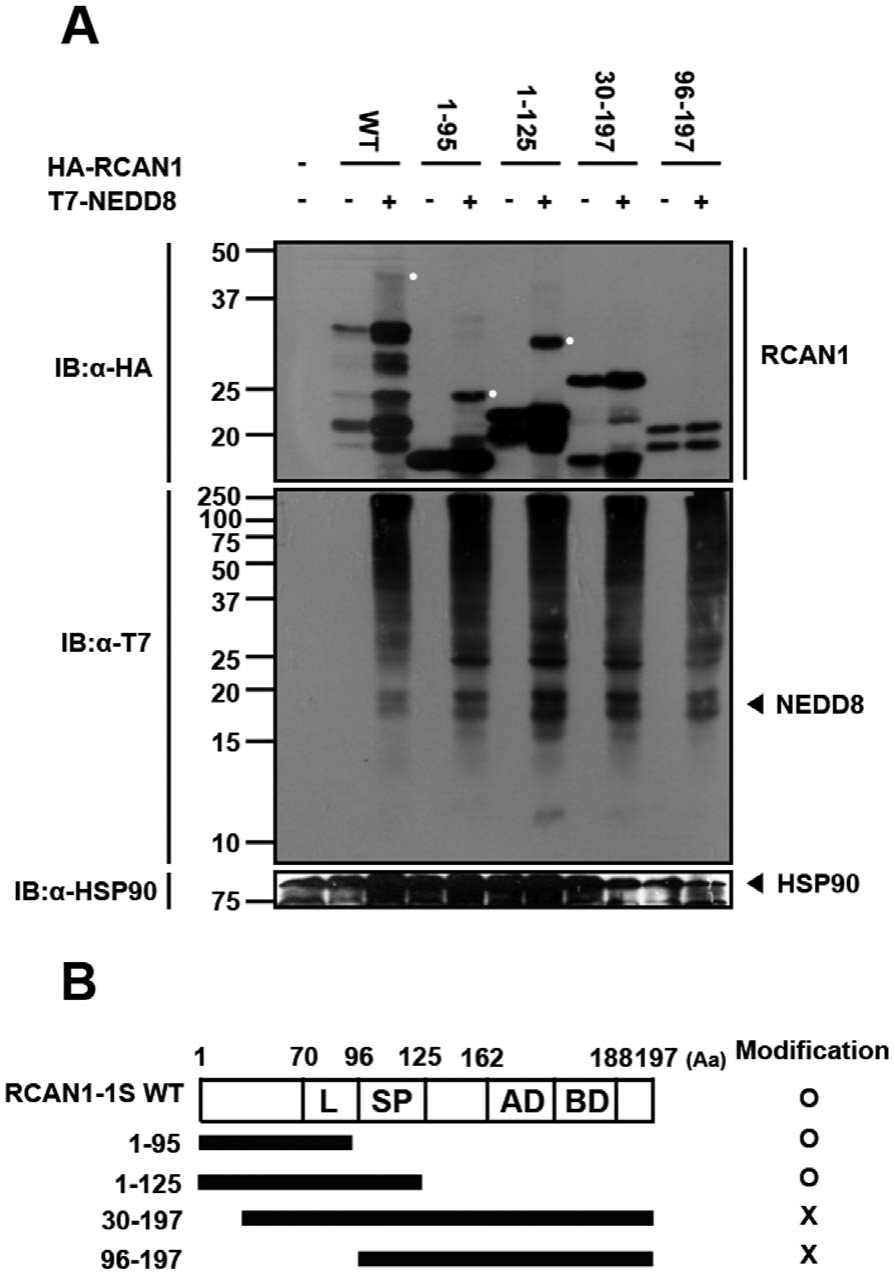
NEDD8 is covalently conjugated to the N-terminal region of RCAN1. (A) Where indicated, HEK293 cells were transfected for 24 h with HA-tagged wild-type RCAN1, RCAN1^1–95^, RCAN1^1–125^, RCAN1^30–197^ or RCAN1^96–197^ fragment in the presence or absence of T7-tagged NEDD8. Cells were lysed with lysis buffer containing 8 M urea and immunoblot analyses were performed using HA antibody. (B) Diagram of full-length RCAN1-1S (WT), its truncation mutants, and a summary of the covalent NEDD8 conjugation assay results. RCAN1 consists of an N-terminal amphipathic leucine repeat (L), a 31 amino acid central span containing serine-proline repeats (SP), and a cluster of acidic (AD) and basic (BD) amino acids.

To identify the RCAN1 region(s) responsible for the NEDD8 association, several HA-tagged truncation mutants of RCAN1 (RCAN1^1–95^, RCAN1^1–125^, RCAN1^30–197^, and RCAN1^96–197^) were generated and evaluated for neddylation. HEK293 cells were co-transfected with plasmids encoding wild-type NEDD8 plus the indicated truncation mutant. Cells were lysed in 8 M urea lysis buffer and analyzed by immunoblot. Western blot analyses with HA antibodies shows that NEDD8 covalently binds the RCAN1^1–95^ and RCAN1^1–125^ mutants as well as wild-type RCAN1 (Fig. 2A). However, the RCAN1^30–197^ and RCAN1^96–197^ mutants were not neddylated (Fig. 2A). These data suggest that amino acids 1–125 of RCAN1 are important for the NEDD8 association (Fig. 2B).

**Figure 3:**
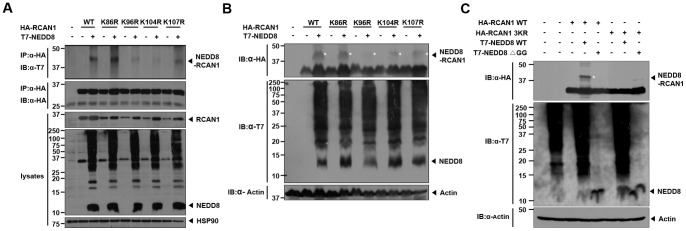
Mapping of NEDD8-targeting site(s) within the RCAN1 protein (A) HEK293 cells were transfected with HA-tagged wild-type RCAN1 or its point mutants K86R, K96R, K104R, and K107R in the presence or absence of T7-tagged NEDD8. Immunoprecipitation was performed with the HA antibody and the immunocomplexes were analyzed by western blot with the T7 antibody. (B) Cells were transfected for 24 h with HA-tagged wild-type RCAN1 or the indicated RCAN1 single-point-mutants in the presence or absence of T7-tagged NEDD8. Cells were lysed with 8 M urea-containing lysis buffer and immunoblot analyses were performed with the HA antibody. (C) HEK293 cells were transfected for 24 h with T7-tagged wild-type NEDD8 (WT), its conjugation-defective mutant (ΔGG), HA-tagged wild-type RCAN1, or RCAN1-3KR mutant alone or in combination and lysed in buffer containing 8 M urea. Immunoblot assays of cell lysates were performed with the HA antibody.

The RCAN1^1–125^ region contains four lysine residues that may be potentially neddylated (K86, K96, K104, and K107). To identify the exact NEDD8 modification site(s), we mutated each these lysine residues to arginine (K86R, K96R, K104R, and K107R) and analyzed neddylation of these RCAN1-point-mutants using *in vivo* neddylation assays. Cell lysates were immunoprecipitated with 1% NP-40 buffer and a strong band of mono-neddylated RCAN1 (∼41 kDa) was observed in cells transfected with wild-type RCAN1 and the RCAN1-K86R mutant, but not in cells transfected with the RCAN1-K96R, -K104R, or -K107R mutants (Fig. 3A). We further confirmed these results by performing the *in vivo* neddylation assays after the cells were lysed in buffer containing 8M urea (Fig. 3B). These results suggest that NEDD8 is conjugated to RCAN1 at multiple sites, including lysines 96, 104 and 107. Alternatively, RCAN1 may be mono-neddylated at one of these lysine residues and the proper conformation of the nearby NEDD8-targeting domain is essential for the reaction to proceed. To determine if all three lysine residues are required for RCAN1-NEDD8 modification, we generated a triple mutant of RCAN1 (RCAN1-3KR) in which K96, K104, and K107 are mutated to arginine and evaluated if it was still modified by NEDD8. The mono-neddylated RCAN1 band was not observed in western blot analyses of cell lysates transfected with RCAN1-3KR and prepared with 8 M urea buffer (Fig. 3C). Taken together, these results suggest that K96, K104, and K107 of RCAN1 are required for neddylation.

### NEDD8 Increases RCAN1 Protein Stability

**Figure 4:**
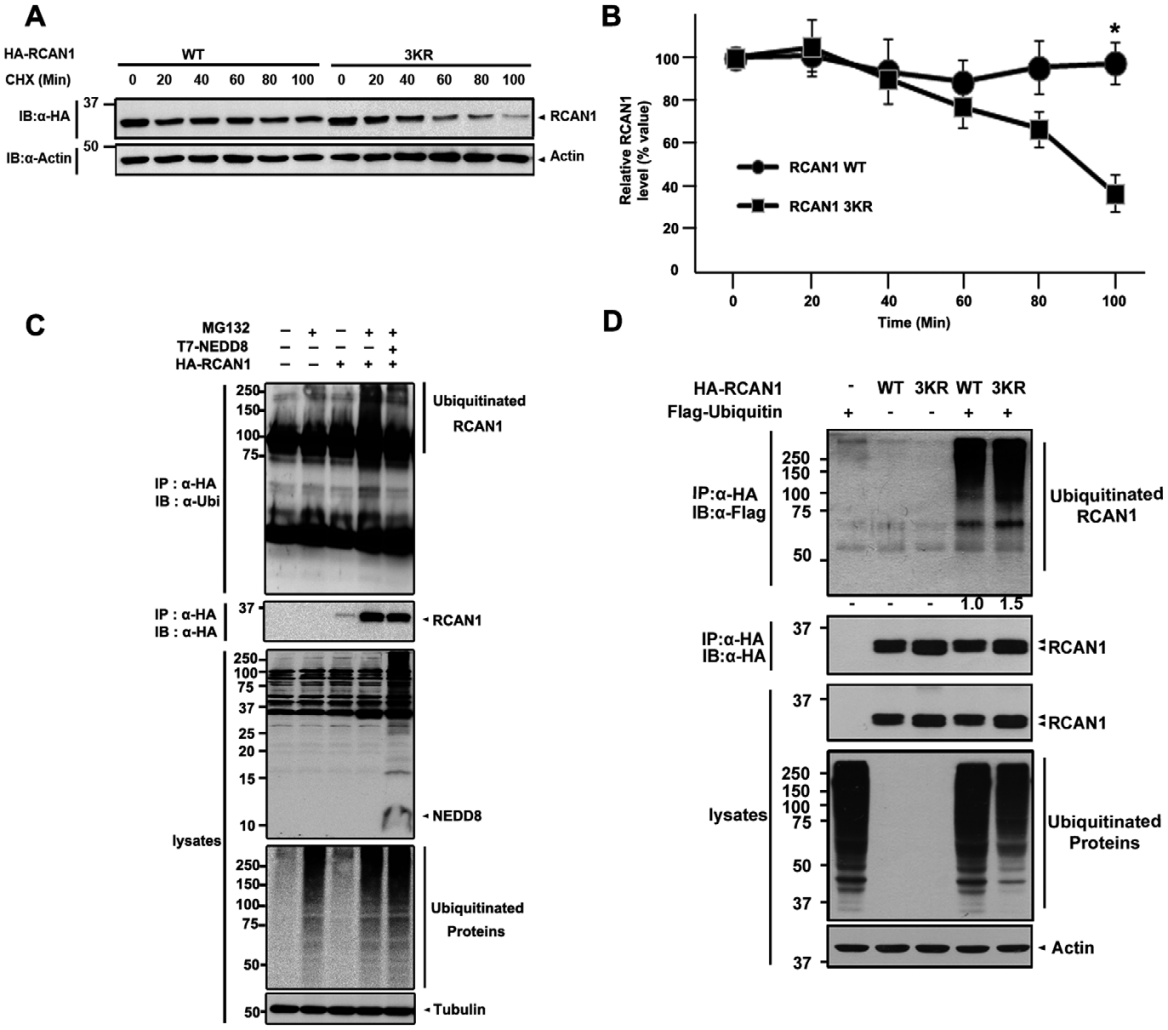
NEDD8-conjugation increases RCAN1 protein stability by inhibiting RCAN1 ubiquitination. (A) Where indicated, HEK293 cells were transfected for 24 h with T7-NEDD8 (NEDD8-WT), and either HA-tagged wild-type RCAN1 or its conjugation-deficient mutant (RCAN1-3KR). Cells were treated with 40 µg/ml cyclohexamide (CHX) for the indicated times and harvested in PBS. The RCAN1 level of each sample was determined by western blot analyses with HA antibody. The data are representative of three independent experiments. (B) The relative RCAN1 protein level was quantified using the Multi Gauge V3.1 program. (C) HEK293 cells were transfected for 24 h with either HA-tagged RCAN1 alone or together with T7-tagged NEDD8, and treated for 6 h in the presence or absence of 10 µM MG132. Cell lysates were immunoprecipitated with the HA antibody, followed by immunoblotting with the ubiquitin antibody. To evaluate expression of T7-NEDD8, HA-RCAN1, and endogenous ubiquitin, cell lysates were analyzed by immunoblotting with anti-T7, anti-HA, or anti-ubiquitin, respectively. (D) HEK293 cells were transfected for 24 h alone or in combination with Flag-ubiquitin, HA-tagged RCAN1-WT, or RCAN1-3KR (MT) and cultured for 6 h in the presence of 10 µM MG132. Total cell lysates and HA immunoprecipitates were probed with Flag or HA antibodies. The values below the top panel indicate the band intensity ratio of ubiquitinated RCAN1 to overexpressed ubiquitin. Band intensities were measured using the Multi Gauge V3.1 software (*, *p*<0.05).

Next, we investigated how NEDD8-modification affects the biochemical properties of RCAN1 protein. In order to compare the protein stability of wild-type RCAN1 with the conjugation-resistant RCAN1 mutant, HEK293 cells were transfected with either HA-tagged wild-type RCAN1 or the RCAN1-3KR mutant, and then incubated with 40 µg/ml cycloheximide for the indicated times. Western blot analyses with the HA antibody and protein band quantification using the Multi Gauge V3.1 program showed that the steady state level of RCAN1-3KR protein is much less than that of wild-type RCAN1 (Fig. 4A and B). Moreover, measurement of the half-life of RCAN1 using cycloheximide revealed that the RCAN1-3KR mutant is degraded more rapidly than wild-type RCAN1 (Fig. 4A and B). These data suggest that neddylation enhances the stability of RCAN1.

To analyze the molecular mechanism responsible for the increased stability of neddylated RCAN1, we first checked whether neddylation affects the extent of RCAN1 ubiquitination. When the cells were pretreated with the proteasomal inhibitor MG132, RCAN1 levels significantly increased (Fig. 4C), consistent with the previous report that RCAN1 stability is mainly regulated through the UPS [14]. Next, we compared the extent of RCAN1 ubiquitination in the absence or presence of NEDD8. As shown in Fig. 4C, the increased RCAN1 ubiquitination induced by MG132 addition was considerably reduced in cells transfected with NEDD8. These data suggest that neddylation inhibits RCAN1 ubiquitination, resulting in the increased RCAN1 levels. This hypothesis is further supported by the finding that ubiquitination of the RCAN1-3KR mutant increases by greater than 50% in the presence of MG132 (Figure 4D). Overall, our results indicate that neddylation increases the level of soluble RCAN1 and this is likely due to decreased RCAN1-ubiquitination and subsequent proteasomal cleavage.

### Neddylation Increases Cytosolic and Nuclear RCAN1 Levels

**Figure 5:**
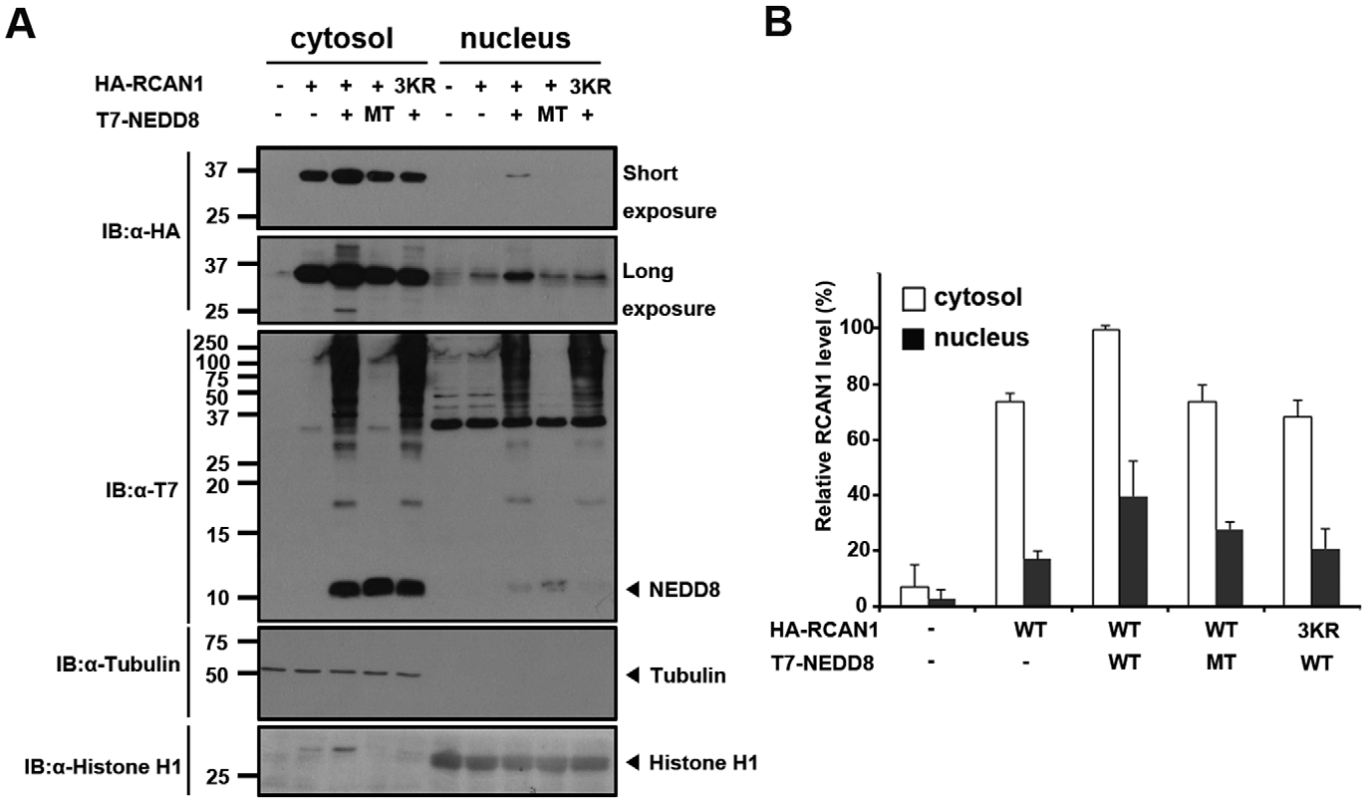
Neddylation increases cytosolic and nuclear RCAN1 levels. (**A**) HEK293 cells were transfected for 24 h with HA-tagged wild-type RCAN1 or the RCAN1-3KR mutant, as indicated. Cell lysates were fractionated into nuclear and cytoplasmic fraction and immunoblot assays were performed with HA or T7 antibodies. The purity of each fraction was confirmed by immunoblotting with α-tubulin (cytosolic marker) or histone H1 (nuclear marker). (**B**) The graph indicates the relative levels of RCAN1 measured using the Multi Gauge V3.1 program and calculated by dividing the HA-RCAN1 band intensity alone by the HA-RCAN1 plus T7-NEDD8 band intensity.

Next, we examined whether covalent NEDD8 attachment changes the intracellular localization of RCAN1. HEK293 cells were transfected with HA-tagged wild-type RCAN1 or the RCAN1-3KR mutant alone or together with either T7-tagged wild-type NEDD8 or NEDD8-ΔGG. Cells were harvested and separated into cytosolic and nuclear fractions. Western blot analyses with HA antibodies showed that cells transfected with wild-type RCAN1 alone had cytosolic and nuclear RCAN1 localization, although it was predominantly in the cytosolic fraction (Fig. 5A and B). Co-expression of wild-type RCAN1 and NEDD8 increased RCAN1 in both the cytosolic and nuclear fractions, although it was still mainly in the cytosol (Fig. 5A and B). In addition, this increase was not observed in cells transfected with wild-type RCAN1 plus NEDD8-ΔGG or the RCAN1-3KR mutant (Fig. 5A and 5B). Immunostaining of the cells further supported these findings (data not shown). We found nuclear RCAN1 was slightly increased when cells were co-transfected with wild-type RCAN1 and NEDD8 (data not shown) and this was not observed in the other conditions. These data suggest that RCAN1 neddylation does not remarkably affect RCAN1 intracellular localization.

### NEDD8-conjugation Increases RCAN1-binding to Calcineurin

**Figure 6:**
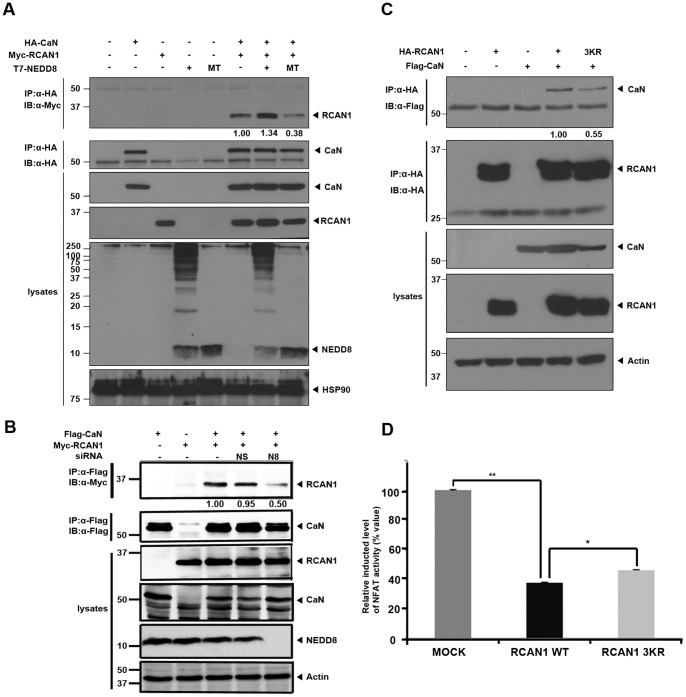
NEDD8-conjugation increases RCAN1 binding to calcineurin and potentiates its inhibitory action of transcriptional NFAT activity. (**A**) HEK293 cells were transfected for 24 h alone or in combination with HA-calcineurin, Myc-RCAN1, or T7-NEDD8. Immunoprecipitation was performed with HA antibodies, followed by immunoblotting with the HA, Myc, or T7 antibodies, as indicated. (**B**) Cells were transfected for 24 h with either NEDD8-specific siRNA (N8; 60 nM) or non-specific control siRNA (NS; 60 nM). Cells were transfected for another 24 h with Myc-RCAN1 in the presence or absence of HA-calcineurin. Cells were lysed in buffer including 1% NP-40, and immunoprecipitation was performed with the HA antibody. Immunoblot analyses of the HA-immunocomplexes were performed with HA, Myc, or NEDD8 antibodies, as indicated. (**C**) HEK293 cells were transfected for 24 h alone or in combination with Flag-calcineurin, HA-tagged wild-type RCAN1, or the RCAN1-3KR mutant. Immunoprecipitation was performed with the HA antibody, followed by immunoblotting with the HA or Flag antibodies. (**D**) HEK293 cells were co-transfected for 24 h with the NFAT firefly luciferase reporter plasmid alone or together with either HA-tagged wild-type RCAN1 or the RCAN1-3KR mutant, as indicated. Cells were lysed and analyzed using the dual-luciferase reporter assay system. The luminescence of each sample was plotted (n = 3; *, *p*<0.05, **, *p*<0.005).

Next, we checked whether neddylation affects the inhibitory action of RCAN1 to calcineurin. HEK293 cells were transfected alone or in combination with Myc-RCAN1, HA-calcineurin, and T7-tagged wild-type NEDD8 or conjugation-defective NEDD8-ΔGG and lysed with 1% NP40 buffer. The RCAN1-calcineurin complex was examined by immunoprecipitation of cell extracts with HA antibodies followed by immunoblot with Myc antibodies. As expected, RCAN1 and calcineurin binding is enhanced by 30% in the presence of wild-type NEDD8, but not with NEDD8-ΔGG. The conjugation-defective mutant decreased the RCAN1-calcineurin complex to well below control levels (Fig. 6A). These data suggest that RCAN1-neddylation enhances formation of the RCAN1-calcineurin complex and under normal growth condition, endogenous NEDD8 significantly contributes to complex formation through RCAN1-modification. These findings were further supported by the observation that knock-down of endogenous NEDD8 specifically decreases RCAN1-calcineurin interaction (Fig. 6B). As a control, we evaluated the possibility of a potential interaction between calcineurin and NEDD8. After cells were co-transfected with T7-NEDD8 and HA-RCAN1, immunoblot analyses of the T7 immunocomplexes with HA antiserum revealed that only NEDD8 binds to calcineurin. However, after cells were transfected with Flag-calcineurin and T7-NEDD8, immunoblot analyses with Flag antibodies of cell lysates prepared using 8M urea-containing lysis buffer detected no upper-shifted band of calcineurin (Figure S1). Thus, our results suggest that NEDD8 binds calcineurin without covalently modifying this protein. These results suggest that calcineurin does not bind NEDD8 directly, and the apparent interaction between NEDD8 and calcineurin may occur indirectly through neddylated RCAN1 (Figure S1). Furthermore, after cells were co-transfected with Flag-calcineurin together with either wild-type RCAN1 or RCAN1-3KR, we compared the level of RCAN1 and calcineurin binding. As shown in Fig. 6C, compared with wild-type RCAN1, cells expressing the RCAN1-3KR mutant showed a 40% reduction in RCAN1 binding to calcineurin (Fig. 6C). Taken together, these data suggest that covalent NEDD8 conjugation increases RCAN1-calcineurin binding, likely as a consequence of enhanced RCAN1 protein stability.

### Neddylation Potentiates the Inhibitory Action of RCAN1 on NFAT Signaling

The phosphatase calcineurin activates NFATc by dephosphorylation [7]. Activated NFATc translocates into the nucleus and up-regulates target gene expression that in turn stimulates cell growth and differentiation. RCAN1 suppresses NFAT-mediated downstream signaling via inhibition of calcineurin. Therefore, we determined whether RCAN1-neddylation also affects NFAT activity and its downstream signaling. After transfection with the NFAT-luciferase reporter plasmid alone or together with either wild-type RCAN1 or the neddylation-defective RCAN1-3KR mutant, cells were treated with the calcium ionophore ionomycin to stimulate Ca^2+^-dependent calcineurin and NFAT activity (Fig. 6D). NFAT-luciferase reporter assays revealed that compared to the control sample with reporter alone, the presence of wild-type RCAN1 inhibits NFAT activity by greater than 60% (Fig. 6D). Compared to wild-type RCAN1, the RCAN1-3KR mutant increased NFAT activity (Fig. 6D). These results indicate that neddylation potentiates the negative effect of RCAN1 on NFAT activity and downstream signaling.

### Decreased Endogenous RCAN1 Results in Reduced Neddylation

**Figure 7:**
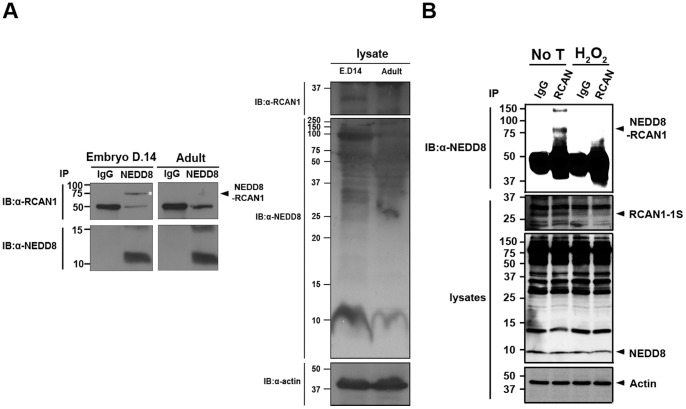
*In vivo* RCAN1-neddylation in mouse brain and after oxidative stress. (**A**) Brains from adult and embryonic day 14 C57BL/6 mice were lysed in RIPA buffer. Immunoprecipitation was performed with either IgG (rabbit) or NEDD8 antiserum followed by western blotting with the RCAN1 antibody. Equal loading of the samples was confirmed by immunoblotting with the actin antibody. (**B**) Hydrogen peroxide treatment induced covalent conjugation of NEDD8 to endogenous RCAN1 in HEK293 cells. Where specified, cells were treated for 2 h with vehicle or 10 µM hydrogen peroxide. Cell lysates were subjected to immunoprecipitation with the RCAN1 antibody, followed by immunoblotting with the NEDD8 antibody. Actin served as a loading control.

In order to verify that RCAN1-neddylation occurs physiologically, we examined whether endogenous RCAN1 is neddylated in the mouse brain. Brains from C57BL/6 adult and embryonic day 14 mice were isolated and evaluated for RCAN1-neddylation. Western blot analyses with the RCAN1 and NEDD8 antibodies showed that endogenous RCAN1 and NEDD8 protein levels are higher in embryonic brain than the adult brain (Fig. 7A). Moreover, co-immunoprecipitation of cell lysates with the RCAN1 antibody followed by immunoblot with the NEDD8 antibody revealed binding between these two proteins occurs in the embryonic brain, but not in the adult brain (Fig. 7A). These data suggest that the increase of endogenous RCAN1 coincides with increased RCAN1-neddylation. Interestingly, the molecular weight of neddylated-RCAN1 (∼78 kDa) was larger than we observed in the samples after DNA overexpression. However, RCAN1 stability correlated well with neddylation of endogenous RCAN1. This result suggests that endogenous RCAN1 may be differentially modified and perhaps is poly-neddylation, rather than mono-neddylation.

To confirm the physiological relevance of *in vivo* RCAN1-neddylation, we examined whether exogenous stimuli that regulate endogenous RCAN1 affect RCAN1-neddylation. Based on reports that oxidative stress induces the ubiquitination and proteasomal degradation of RCAN1-1S in mammalian cells [15], [16], we assessed the effect of hydrogen peroxide treatment on endogenous RCAN1 levels in HEK293 cells. As shown in Fig. 7B, the addition of hydrogen peroxide significantly decreased endogenous RCAN1. In addition, we found that the decreased RCAN1 level in response to H_2_O_2_ is concurrent with a remarkable decrease in RCAN1-neddylation (Fig. 7B). Interestingly, the molecular weight of the neddylated RCAN1 band induced by hydrogen peroxide was the same size as observed in the embryonic mouse brain (Fig. 7A and B), supporting the idea that endogenous RCAN1 may be a target of poly-NEDD8 conjugation. These data also suggest the NEDD8-conjugation to RCAN1 is not an artifact of ectopic DNA expression. Depending on the developmental stages or cellular stress, neddylation affects RCAN1-mediated physiological actions by down-regulating RCAN1 protein levels in mammalian cells.

## Discussion

Studies have shown that RCAN1 function is modulated by a number of post-translational modifications and non-covalent interactions with other proteins. For example, RCAN1 activity is primarily regulated through phosphorylation. Glycogen synthase kinase-3 [17], MEKK3 [18], BMK1 [19], Dyrk1A [20], and NF-κB-inducing kinase [21] phosphorylate RCAN1 and alter its biochemical properties, including stability and intracellular localization. In addition to calcineurin, RCAN1 binds 14-3-3ε/ξ [19], Raf-1 [22] and Tollip [23]. Moreover, the RCAN1 protein is regulated by various degradation pathways, including the UPS and lysosomal pathways [14], [24], [25]. Specifically, FBW7 and NEDD4-2 were reported to act as ubiquitin E3 enzymes that ubiquitinate RCAN1 and target it for proteasomal degradation [26], [27]. Here, we report a novel mode of RCAN1 modulation via NEDD8. Our data show that RCAN1 can be neddylated in cultured cell lines to increase RCAN1 protein stability and subsequently potentiate its inhibitory action toward calcineurin and downstream NFAT signaling.

Although RCAN1 appears to be mono-neddylated in HEK293 cells, mapping of targeting sites revealed that three lysine residues are required for proper RCAN1-neddylation. These results suggest that RCAN1 is mono-neddylated randomly at one of these lysine residues. Alternatively, a single lysine residue is neddylated but the nearby lysine residues are critical for proper NEDD8 modification to proceed. Similar phenomena have been reported for ubiquitin-like SUMO-modification in many other targets. For example, a region downstream of the target lysine residues is required for SUMOylation of the Kruppel zinger finger protein ZNF146 [28]. In addition, multiple mutations at adjacent lysine resides near the direct SUMO-1 targeting site affects the overall folding of MMLV capsid protein and indirectly blocks its modification [29].

Similar to protein ubiquitination, NEDD8 is conjugated to the target protein via an isopeptide bond between the substrate lysine and the C-terminal glycine 76 of NEDD8 [11]. Neddylation consequently triggers diverse changes in the target protein, including conformational changes, cellular localization changes, and modifying protein-protein interactions between its partners [11], [30], [13]. For example, neddylation induces changes in cullin that subsequently inhibits its interaction with CAND1 and stimulates the ubiquitin E3 ligase activity of SCF complexes [31], [32]. NEDD8 conjugation has also been implicated in neurodegenerative disorders, such as in formation of the abnormal protein inclusion bodies in Parkinson disease [33]. Moreover, NEDD8 immunoreactivity is present in brain tissues from patients with various neurodegenerative disorders [34].

As the full name indicates, NEDD8 gene is highly expressed in the embryonic mouse brain and then down-regulated during the brain development [35]. In the same manner, the present data showed that NEDD8 protein is differentially detected in mouse embryo and adult brains, and much enhanced neddylation pattern was observed in embryonic stage. Similar to this pattern, the level of NEDD8-conjugated RCAN1 protein was highly detected in embryonic mouse brain, compared with the adult tissue. Based on the previous report and the current finding we could speculate that highly expressed pattern of NEDD8 may contribute to the increase of RCAN1-neddylation, which eventually leads to more accumulation of RCAN1 in embryonic stage. Or else, highly expressed RCAN1 level and/or more stable RCAN1 form at early developmental period would be more modified through NEDD8, which causes the increase of RCAN1 stability in an additive manner. In addition, although the functional roles of differentially regulated RCAN1 during developmental stages were not explored before, the accumulation of RCAN1 may have a specific role on the mouse brain development. More additional experiments would be required to test whether hypothesis is correct. Furthermore, it would be interesting to further test whether neddylated RCAN1 may have an unspecified functional role that is closely linked to the early stages of mouse brain development.

Interestingly, compared with the observed molecular weight of the neddylated RCAN1 protein in HEK293 cells, we observed an upper shifted band corresponding to modified RCAN1 in mouse brain. One explanation is that neddylated RCAN1 may form a complex with additional proteins, such as calcineurin, in mouse brain. Alternatively, although the target has similar functional alterations after modification, the reaction mechanisms for covalent RCAN1-neddylation may be different *in vivo* versus ectopic DNA transfection condition. Similar patterns have been observed in many other targets subjected to SUMO-modification, including Ubc9 [36], FOXc1 [37], and zinc finger protein ZNF131 [38]. For example, HIF1α is poly-SUMOylated when exogenously transfected but is mono-SUMOylated during hypoxia [39].

Neddylation requires the coordinated action of APP-BP1/Uba3 (a heteromeric E1-like enzyme) and UbcH12 (an E2-like enzyme). The NEDD8 E3 ligase for many neddylation targets has yet to be identified. Nevertheless, several enzymes are known to directly mediate the neddylation of some targets, although it is not clear whether a ligase is required in all reactions. These include Rbx1 and/or Dcn1 for the modification of cullin, Parc, and Cul7 [40]; Mdm2 for p53, p73, and Mdm2 itself [41], [42]; c-Cbl for EFGR [11]; and TRIP12 for APP-BP1 [43]. To verify RCAN1-neddylation and determine if an E3 is required, we performed *in vitro* neddylation assays in the presence of E1 and E2, but in the absence of E3. Recombinant GST-fused proteins, including GST-RCAN1 and GST-NEDD8 were purified from bacteria and incubated with the recombinant E1 (APPBP1-Uba3) and E2 enzyme (UbcH12). Western blot analyses with RCAN1 antibodies showed no obvious RCAN1 neddylation band (Figure S2). This result suggests that an unknown E3 enzyme(s) is necessary for complete RCAN1 neddylation. Alternatively, a critical factor(s) for RCAN1 neddylation may be missing in the *in vitro* system. Overall, these results indicate that RCAN1 is not neddylated *in vitro* in the presence of E1 and E2 alone.

Several proteins are substrates for both NEDD8 and ubiquitin. For example, the cullin family of proteins are targets of covalent NEDD8-conjugation and act as scaffold components of ubiquitin E3 ligase for multiple substrates, such as EGF receptor, IκB, and HIF-1α [44]–[46]. In addition, HIF-1α is modified through ubiquitin and NEDD8. The VBC/Cul-2 complex acts as an ubiquitin E3 ligase and mediates HIF-α ubiquitination in the nuclear compartment when cells are exposed to normal oxygen tension. HIF-α is then exported to the cytoplasm and degraded through proteasomal machinery [47]. HIF-1α is also covalently modified via NEDD8, which increases the HIF-1α protein level in normoxia as well as in hypoxia [48]. Similar to these proteins, RCAN1 is a target of ubiquitination as well as neddylation. NEDD8- and ubiquitin-conjugation to a common protein target may occur through the same or distinct lysine residue(s), suggesting that they may compete with each other, or conjugate either sequentially or simultaneously.

Several studies indicate that NEDD8 may play a role in proteolysis through the UPS [11], [13]. For example, NEDD8 is conjugated to Cul-1, which stimulates the ubiquitin ligase activity of SCF^Skp2^ [49], [50] and SCF^β-TrCP^ [51]. Mechanistically, NEDD8 appears to stimulate SCF complex activity through a Cul-1 conformational change that promotes efficient formation of an E3 complex. Cul-2 neddylation also facilitates the polyubiquitination and subsequent proteasomal degradation of HIF-1α [47]. Interestingly, RCAN1 neddylation significantly inhibits RCAN1 ubiquitination. Based on our finding that the ubiquitination of the RCAN1-3KR mutant is greatly increased, these data suggest that RCAN1 ubiquitination and neddylation compete with each other. Alternatively, accumulation of neddylated RCAN1 blocks activity of the enzyme required for RCAN1 ubiquitination and thus inhibits its proteasomal degradation. We previously reported that RCAN1 protein levels are regulated by UPS [14]. It was also found that SCF^β-TrCP^-mediated poly-ubiquitination of RCAN1 occurs upon oxidative stress [15]. Moreover, we observed that STAT2 enhances RCAN1 ubiquitination through the recruitment of ubiquitin E3 ligase FBW7 [52]. It would be interesting to test the binding affinity of neddylated RCAN1 to the known ubiquitin E3 ligases of RCAN1, including FBW7, SCF^β-TrCP^, and NEDD4-2, to validate our hypothesis.

Together, these results suggest that NEDD8 influences diverse RCAN1-mediated cellular processes by positively affecting RCAN1 protein stability.

## Materials and Methods

### Materials

Dulbecco’s modified Eagle’s medium (DMEM), fetal bovine serum (FBS), LipofectAMINE PLUS™ reagent were purchased from Life Technologies (Grand Island, NY, USA). Protein A-Sepharose was obtained from GE Healthcare Life Sciences (Piscataway, NJ, USA). The goat anti-rabbit and anti-mouse IgG secondary antibodies (horseradish peroxidase-conjugated) were purchased from Zymed Laboratories (San Francisco, CA, USA). The enhanced chemiluminescence (ECL) reagent was purchased from Perkin-Elmer Life and Analytical Sciences (Waltham, MA, USA). MG132 was purchased from A. G. Scientific (San Diego, CA, USA). The HA, GFP, GST, Hsp90, ubiquitin, α-tubulin, histone H1, and Myc antibodies were purchased from Santa Cruz Biotechnology (Santa Cruz, CA, USA). The Flag antibody and cycloheximide were obtained from Sigma-Aldrich (St. Louis, MO, USA). The rabbit polyclonal T7 antibody was purchased from Covance (Princeton, NJ, USA), and the mouse monoclonal T7 antibody was from Novagen (Madison, WI, USA). The polyclonal NEDD8 antibody was purchased from Cell Signaling (Beverly, MA, USA).

### DNA Constructs and RNA Interference

The mammalian expression vectors for HA-tagged human wild-type RCAN1 (RCAN1-1S) and HA-tagged wild-type calcineurin A were kindly provided by S. de la Luna (Genomics Regulation Center, Barcelona, Spain) and B. A. Rothermel (University of Texas Southwestern Medical Center, Dallas, TX, USA), respectively. Plasmids encoding GFP-tagged NEDD8 and NEDD8-ΔGG were provided by C.Y. Choi (Sungkyunkwan University, Suwon, Korea). Plasmids encoding T7-tagged wild-type NEDD8 and its conjugation-defective mutant, NEDD8-ΔGG, were kindly provided by M. Ohh (University of Toronto, Toronto, Canada). The NFAT-driven reporter plasmid (pGL-IL2-Luc) was kindly provided by G.R. Crabtree (Stanford University School of Medicine, Stanford, CA, USA). The NEDD8 siRNA duplex sequence was 5′-CAUAAUGAGGCAGCAUCAUAUA-3′ and 5′-UAUAUGAUGCCUCAUUAUG-3′ (Bioneer, Daejeon, Korea). RCAN1 mutants having single or multiple point mutations [RCAN1-K86R, RCAN1-K96R, RCAN1-K104R, RCAN1-K107R, and RCAN1-3KR (K96, K104, and K107 are mutated to R)] were generated from HA-tagged full-length RCAN1 using the QuikChange® XL Site-Directed Mutagenesis Kit (Agilent Technologies, Santa Clara, CA USA), according to the manufacturer’s instructions. Bacterial expression vectors encoding GST-fused RCAN1 and NEDD8 were constructed by PCR and subcloning into pGEX-4T1 (GE Healthcare Life Sciences). All constructs were confirmed by DNA sequencing (Cosmogenetech, Seoul, Korea).

### Cell Culture and DNA Transfection

Human embryonic kidney cells (HEK293) and African Green Monkey fibroblast-like kidney cells (COS-7) were cultured in DMEM containing 10% FBS, 100 units/ml of penicillin and 100 µg/ml streptomycin. Cells were grown at 37°C in 5% CO_2_. All DNA transfections were performed using LipofectAMINE PLUS reagents (Life Technologies), according to the manufacturer’s protocol.

### Immunoprecipitation and Immunoblot Assay

Cells were rinsed twice with ice-cold phosphate-buffered saline (PBS) and scraped in lysis buffer [50 mM Tris (pH 7.5), containing 1.0% Nonidet P-40, 150 mM NaCl, 10% glycerol, 1 mM Na_3_VO_4_, 1 µg/ml leupeptin, 1 µg/ml aprotinin, 1 mM EGTA, 1 mM EDTA,10 mM NaF, and 0.2 mM phenylmethylsulfonyl fluoride]. Cell lysates were collected after a 20 min centrifugation at 13,000×g at 4°C. One microgram of each antibody was incubated with 0.5 to 1 mg of cell extracts prepared in cell lysis buffer overnight at 4°C. Next, 50 µl of protein A-sepharose beads (1∶1 suspension) was added and incubated for 2 h at 4°C with gentle rotation. Beads were pelleted and washed extensively with cell lysis buffer. The immunocomplexes were dissociated by boiling in SDS-PAGE sample buffer, separated on SDS-PAGE gels, and transferred onto nitrocellulose membranes (Whatman, Piscataway, NJ, USA). Membranes were blocked for 1 h at room temperature in 5% nonfat dry milk in TBST buffer [20 mM Tris (pH 7.5), 137 mM NaCl, and 0.1% Tween 20] and incubated overnight at 4°C in 3% nonfat dry milk TBST buffer with the appropriate primary antibody. Membranes were washed three times in TBST, followed by 1 h incubation at room temperature with the appropriate secondary IgG-coupled horseradish peroxidase antibody. The membranes were washed three times with TBST and visualized with ECL reagent.

### Immunohistochemistry

Cells were fixed in 3.7% formaldehyde in PBS solution, washed twice with PBS, and permeabilized in 0.2% Triton X-100 in PBS. After permeabilization, cell were blocked with 1% bovine serum albumin and incubated with the primary antibodies. After two PBS washes, cells were incubated with TRITC- or FITC-conjugated secondary antibodies. The cells were again washed in PBS, and mounted in Slow Fade Light Anti-fade reagent with DAPI (Invitrogen, Carlsbad, CA, USA) Immunostained cells were observed using a Carl Zeiss LSM-510 META confocal microscope.

### *In vitro* Neddylation Assay

Recombinant GST-fused proteins, including GST-RCAN1 and GST-NEDD8, were purified from *E. coli*. For *in vitro* neddylation assays, 10 ng of GST or GST-RCAN1 was incubated for 2 h at 37°C with 200 ng GST-NEDD8, 500 ng E1 (APPBP1-Uba3; Enzo Life Sciences, Plymouth Meeting, PA, USA), and 200 ng GST-E2 (UbcH12; Enzo Life Sciences) in a total reaction volume of 10 µl [40 mM Tris-HCl (pH 7.4), 5 mM MgCl_2_, 2 mM ATP, 2 mM dithiothreitol]. The reaction was stopped by the addition of SDS sample buffer and samples were subjected to SDS-PAGE. Covalent NEDD8 modification of RCAN1 was detected by western blot using GST or NEDD8 antibodies.

### Preparation of Cytosolic and Nuclear Fractions

Cells were washed with ice-cold PBS and re-suspended in hypotonic buffer [10 mM HEPES (pH 7.9), 1.5 mM MgCl_2_, 10 mM KCl] supplemented with protease inhibitors (including dithiothreitol, aprotinin, and leupeptin) and incubated for 30 min on ice. Next, the cells were lysed with a disposable syringe, followed by centrifugation at 1,000×*g* for 15 min at 4°C. The resulting supernatant is the cytosolic fraction. The nuclear pellet fractions were washed with hypotonic buffer and lysed with 1.0% NP-40 lysis buffer. Supernatants from each fraction were collected after centrifugation at 15,000×*g* for 15 min at 4°C.

### Luciferase Reporter Assay

HEK293 cell were co-transfected with the firefly luciferase reporter plasmid (pGL-IL2-Luc) containing a synthetic NFAT binding site and interleukin-2 (IL-2)-minimal promoter and the pRL-TK plasmid constitutively expressing *Renilla* luciferase (to normalize for transfection efficiency). A subset of cells was co-transfected with HA-tagged wild-type RCAN1 or RCAN1-3KR mutant plasmids. Twenty-four hours after transfection, cells were lysed and analyzed using the dual-luciferase reporter assay system (Promega Corporation, Madison, WI, USA).

## Supporting Information

Figure S1 **Calcineruin indirectly binds to NEDD8 via RCAN1 in HEK293 cells.** (A) HEK293 cells were transfected with plasmids encoding HA-tagged calcineurin (CaN) and/or T7-tagged NEDD8 for 24 h. Immunoprecipitation (IP) was performed with anti-T7 antibodies, and the immunocomplexes were analyzed by Western blotting with anti-HA or -T7 antibodies. (B) HEK293 cells were transfected with Flag-CaN, T7-tagged wild type NEDD8, or its conjugation-defective mutant (MT) alone or in combination for 24 h, and the cells were lysed with the lysis buffer containing 8 M urea. Immunoblot analysis of cell lysates was performed with anti-Flag antibodies.
(TIF)

Figure S2 **RCAN1 is not modified by NEDD8** ***in vitro*** **in the presence of E1 and E2.** *In vitro* neddylation assay was performed by incubating 10 ng GST or GST-RCAN1 with 200 ng GST-NEDD8, 500 ng recombinant APPBP1-Uba3, and 200 ng GST-UbcH12 for 2 h at 37°C. The reaction products were subjected to western blotting with anti-RCAN1 or anti-NEDD8 antibody.
(TIF)
