# Supplementary figures and images for: Covalent NEDD8 Conjugation Increases RCAN1 Protein Stability and Potentiates Its Inhibitory Action on Calcineurin
Source: PLoS One. 2012 Oct 31;7(10):e48315. doi: 10.1371/journal.pone.0048315 (PMC3485183; doi:10.1371/journal.pone.0048315)

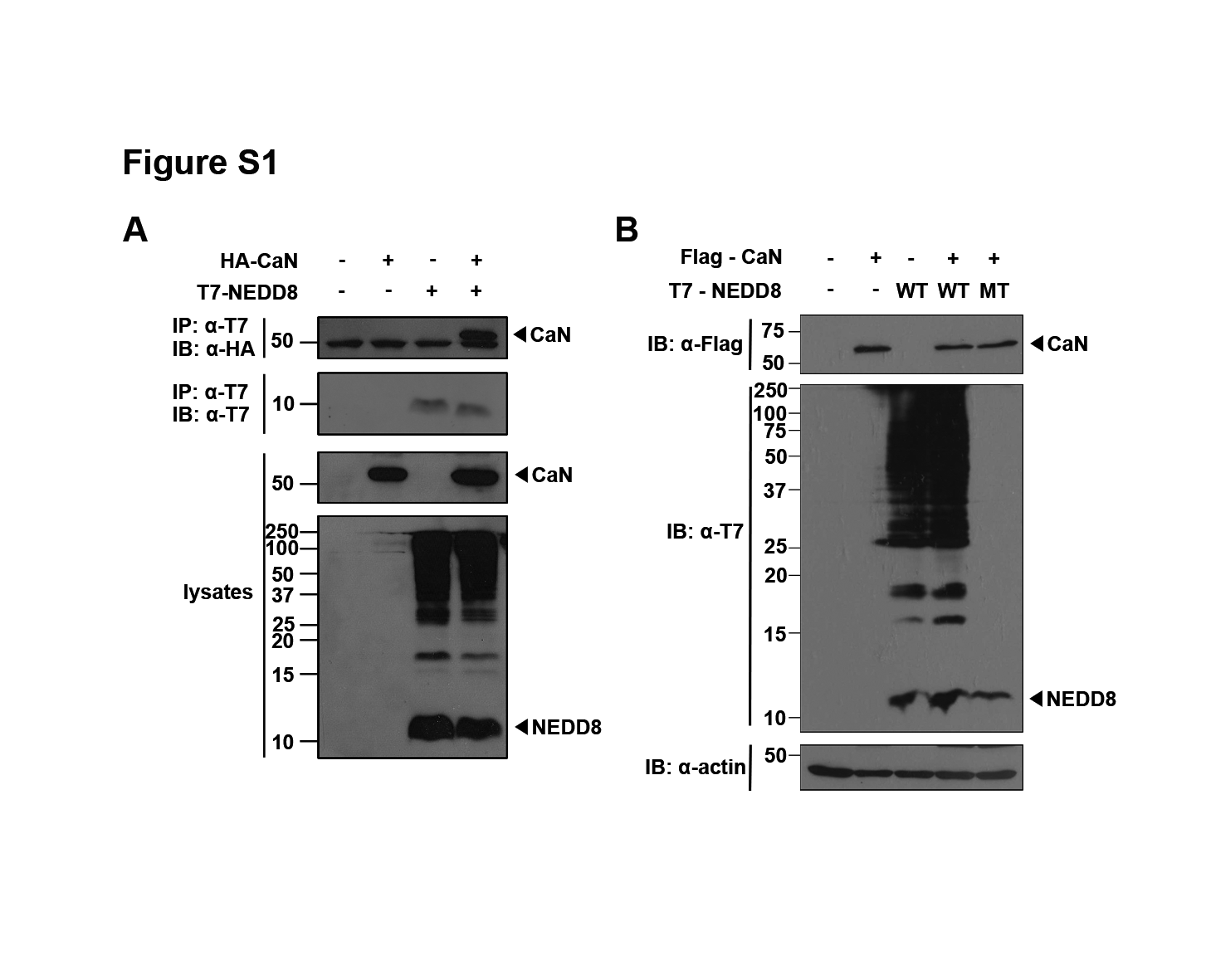

Supplement: Figure S1 — Calcineruin indirectly binds to NEDD8 via RCAN1 in HEK293 cells. (A) HEK293 cells were transfected with plasmids encoding HA-tagged calcineurin (CaN) and/or T7-tagged NEDD8 for 24 h. Immunoprecipitation (IP) was performed with anti-T7 antibodies, and the immunocomplexes were analyzed by Western blotting with anti-HA or -T7 antibodies. (B) HEK293 cells were transfected with Flag-CaN, T7-tagged wild type NEDD8, or its conjugation-defective mutant (MT) alone or in combination for 24 h, and the cells were lysed with the lysis buffer containing 8 M urea. Immunoblot analysis of cell lysates was performed with anti-Flag antibodies. (TIF) [file pone.0048315.s001.tif]

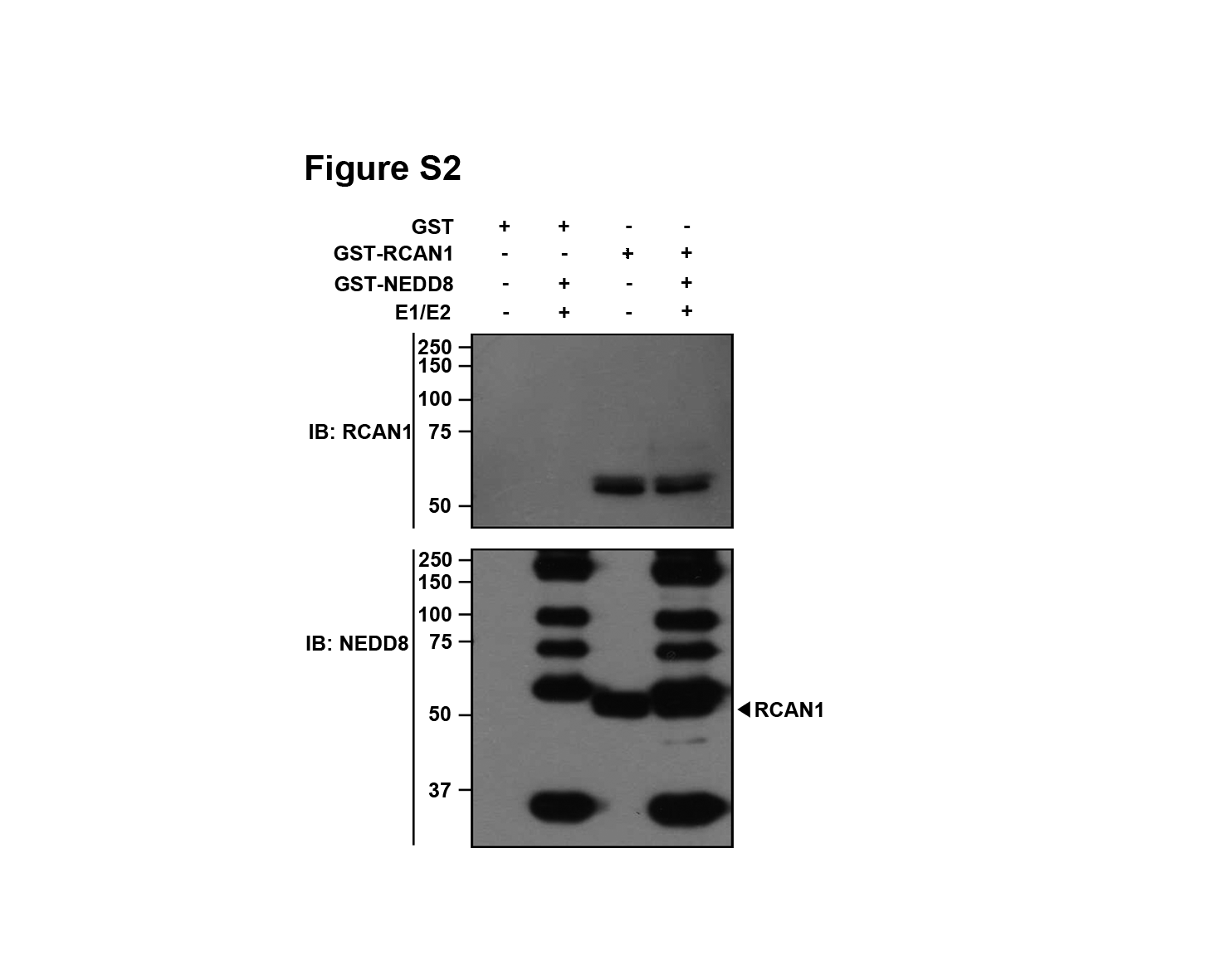

Supplement: Figure S2 — RCAN1 is not modified by NEDD8 in vitro in the presence of E1 and E2. In vitro neddylation assay was performed by incubating 10 ng GST or GST-RCAN1 with 200 ng GST-NEDD8, 500 ng recombinant APPBP1-Uba3, and 200 ng GST-UbcH12 for 2 h at 37°C. The reaction products were subjected to western blotting with anti-RCAN1 or anti-NEDD8 antibody. (TIF) [file pone.0048315.s002.tif]
